# Attachment Insecurity and Altruistic Behavior among Chinese Adolescents: Mediating Effect of Different Dimensions of Empathy

**DOI:** 10.3390/ijerph191610371

**Published:** 2022-08-20

**Authors:** Yangu Pan, Shuang Liang, Daniel T. L. Shek

**Affiliations:** 1 Research Institute of Social Development, Southwestern University of Finance and Economics, Chengdu 611130, China; 2 Department of Applied Social Sciences, The Hong Kong Polytechnic University, Hong Kong SAR, China

**Keywords:** attachment avoidance, attachment anxiety, empathy, altruistic behavior, adolescents, China

## Abstract

Although Western studies showed that attachment insecurity was negatively related to adolescent altruistic behavior, few studies have investigated this issue among Chinese adolescents, and little is known about the mechanisms underlying the impact of attachment avoidance and attachment anxiety on adolescent altruistic behaviors. This study investigated the mediating role of different dimensions of empathy (empathic concern, perspective taking, and personal distress) on the association of attachment avoidance and attachment anxiety with altruistic behavior among Chinese adolescents. A total of 1005 7th and 8th grade Chinese students (*M*_age_ = 12.86 years, *SD* = 0.69) from three middle schools in Chengdu, China completed measures of attachment insecurity, interpersonal reactivity index, and altruistic behavior. Results indicated that attachment avoidance, not attachment anxiety, negatively predicted adolescent altruistic behavior among Chinese adolescents. Moreover, higher attachment avoidance predicted less empathic concern and perspective taking, which in turn predicted less altruistic behavior, while higher attachment anxiety predicted more empathic concern and personal distress, which further predicted more and less altruistic behavior, respectively. These findings highlight the importance of promoting adolescent empathic concern and perspective taking and reducing personal distress to strengthen adolescent altruistic behavior.

## 1. Introduction

Altruistic behavior refers to behavior aiming at increasing the welfare of other individuals [1] and is commonly regarded as a form of prosocial behavior [2]. Altruistic behavior is an evolved human capacity which can confer benefits on both the giver and the receiver. Altruistic behavior plays an important role in maintaining individual health and good interpersonal relationship as well as promoting social harmony [3]. Recently, researchers in personality and social psychology have investigated altruistic behavior from the perspective of attachment theory, and found that individuals with secure attachment showed more altruistic behavior relative to individuals with insecure attachment. Hence, researchers proposed that secure attachment could promote altruistic behaviors toward others in distress [4,5,6]. However, few studies have investigated the mechanisms underlying the association between attachment security (and insecurity) and altruistic behavior, especially in non-Western societies, including China which has roughly one-fifth of the world’s population. Guided by the attachment theory [6], the present study examined whether empathy indexed by empathic concern, perspective taking, and personal distress links attachment insecurity (avoidance and anxiety) to altruistic behaviors among Chinese adolescents.

### 1.1. Attachment Insecurity and Altruistic Behavior

Attachment styles refer to “systematic patterns of expectations, needs, emotions, emotion-regulation strategies, and social behavior” [7] (p. 134) in close relationships with family members, romantic partners, and close friends [8]. Attachment styles are commonly conceived in terms of two dimensions, including attachment-related avoidance and attachment-related anxiety in close relationships [9]. High attachment anxiety reflects an intense fear of being rejected and abandoned, a strong desire for relationship closeness, and high preoccupation with relationship needs [7]. Conversely, high attachment avoidance reflects discomfort with intimacy, dependency, and emotional disclosure in close relationships. A high level on one or both of these dimensions reflects greater attachment insecurity, while lower levels on both dimensions reflect greater attachment security.

Studies showed that individuals with insecure attachment have negative internal working models. In particular, individuals with attachment anxiety have a negative “self-model” with a perception of themselves as unlovable and incompetent, whereas individuals with attachment avoidance have a negative “other-model” with a perception of others as untrustworthy and unreliable [7,10,11]. Moreover, individuals with high attachment anxiety tend to adopt “hyper-activating” attachment strategies—energetic, insistent attempts to obtain care, support, and love from relationship partners—as a means of regulating distress, while individuals with high attachment avoidance tend to rely on “deactivating” attachment strategies, such as suppression of attachment-related thoughts and emotions [8]. Individuals with attachment security have both a positive “self-model” with a perception of themselves as loveable and competent and a positive “other-model” with a perception of others as trustworthy and reliable.

A review study indicated that attachment insecurity (attachment avoidance and attachment anxiety) was closely associated with the negative development of prosocial motives, emotions, and behavior [5]. For example, an experimental study showed that attachment avoidance and attachment anxiety negatively predicted individual altruistic behavior [12]. A survey study also found that higher attachment avoidance was related to less volunteering and less altruistic and exploration-oriented motives for volunteering, while higher attachment anxiety was associated with increased self-enhancing motives for volunteering [13]. Moreover, Erez et al. [14] found attachment insecurities (avoidance and anxiety) made a unique contribution to volunteerism after controlling for positive personality traits including agreeableness and conscientiousness. Westmaas and Silver [15] suggested that attachment anxiety is an important predictor of anxious reaction to victims, while attachment avoidance is a potentially important predictor of the likelihood of supportive responses to victims. With particular reference to adolescents, Allen and Tan [16] argued that adolescents’ attachment surrounds one’s parents, peers and romantic partners. Stern et al. [17] found that secure attachment predicted adolescents’ ability to provide empathic support in close friendships.

### 1.2. Attachment Insecurity and Empathy

Empathy refers to understanding and “feeling with” others’ emotions, and is an essential capacity underlying sensitive care in humans and other species. Eisenberg and Eggum [18] (p. 71) defined empathy as “an affective response that stems from the apprehension or comprehension of another’s emotional state or condition, and which is similar to what the other person is feeling or would be expected to feel”. In real-life situations, it is likely that empathy often turns into sympathy, personal distress, or both (e.g., alternating). According to Eisenberg and Eggum [18] (pp. 71–72), “sympathy is an emotional response, stemming from the apprehension of another’s emotional state or condition, that is not the same as the other’s state or condition but consists of feelings of sorrow or concern for the other”. Others have sometimes labeled sympathy as empathic concern or have included such responses in their definitions of empathy (e.g., [19,20]). In contrast, Eisenberg and Eggum [18] (p. 72) conceived personal distress as “a self-focused, aversive affective reaction to the apprehension of another’s emotion, associated with the desire to alleviate one’s own, but not the other’s distress such as discomfort and anxiety”. In addition, some studies in the literature have argued that perspective taking is an important component of empathy [21]. Perspective taking is the tendency to understand another’s thoughts, feelings, or situation [9,22,23]. It is a cognitive capacity to consider the world from other viewpoints and “allows an individual to anticipate the behavior and reactions of others” [22].

A review suggested that attachment may contribute to individual differences in empathy from infancy through adolescence [24]. Mikulincer et al. [25] showed that attachment avoidance and attachment anxiety were inversely related to empathic concern (i.e., sympathy), and attachment anxiety was positively associated with personal distress. Another study found that insecure attachment styles were negatively associated with empathy in nursing students [26]. Moreover, studies showed that attachment-security priming strengthened empathic reactions and compassion, and inhibited personal distress [12,25]. Recently, a meta-analytic study found that attachment avoidance was negatively associated with empathy, while ambivalent attachment (i.e., attachment anxiety) was not associated with empathy [27].

### 1.3. Empathy and Altruistic Behavior

Although a previous meta-analytic study showed that empathy was positively associated with prosocial behavior [28], two important components of empathy (sympathy and personal distress) were associated differentially with prosocial behavior [29]. Similarly, other studies showed that sympathy tended to be positively related to prosocial behavior, whereas personal distress was negatively related or unrelated to prosocial behavior when the actor could escape contact with the person evoking the distress [19,30]. Theoretically, Batson [19] proposed the “empathy-altruism connection” to explain the relation between empathy and altruistic behavior where viewing another’s distress can evoke a mixture of self-focused distress and other-focused empathy. While researchers agreed that self-focused distress triggers egoistic motives, they debated whether other-focused empathy could trigger a pure altruistic motive. De Waal [31] believed that other-focused empathy (i.e., sympathy) is the intrinsic motivation for altruistic behaviors [32,33]. Additionally, Carlo, Allen and Buhman [34] found that perspective taking was positively related to prosocial behavior.

### 1.4. Attachment, Empathy and Altruistic Behavior in Chinese Culture

Culture might moderate the association between attachment and child empathy [24]. A meta-analytic study found that secure attachment was positively related to empathy (*r* = 0.27) among children and adolescents, and culture is a significant moderator, with larger effect sizes being observed in samples from Asian countries (*N*_sample_ = 14, *r* = 0.33), compared to samples from Western countries (*N*_sample_ = 44, *r* = 0.24) [27]. Additionally, another meta-analytic study found the relation between attachment and empathy in Eastern culture was higher than that in Western culture [35]. Moreover, a meta-analytic study found that empathy was positively associated with prosocial behavior (*r* = 0.38), and cultural background (Western, *N*_sample_ = 38 vs. Chinese, *N*_sample_ = 39) had no moderating effect [36].

Influenced by Confucianism, China is a typical collectivistic culture country. Specifically, in self-construal, Chinese people are interdependent, while Westerners are independent [37]. In emotional expressivity, Chinese people might show less personal distress and empathic concern in response to others’ distress compared with Westerners [38]. In cognitive styles, Chinese tend to think holistically, while Westerners tend to think analytically [39,40]. These culture characteristics could influence the relation between attachment, empathy, and altruistic behaviors among Chinese children and adolescents. Unfortunately, empirical studies on adolescent behavior are unsystematic and inadequate [41,42].

Mesman et al. [43] suggested that incidence of insecure attachment was higher in developing countries due to socioeconomic difficulties. As a result, the variance of insecure attachment may be larger in developing countries (e.g., Chinese culture) than developed countries (e.g., American cultures). Moreover, previous studies suggested that Chinese adolescents (collectivistic culture) have more perspective taking and personal distress, and less empathic concern compared with Western adolescents (individualistic culture) [44,45,46].

Previous studies found that parent–child attachment had a positive effect on prosocial behavior among Chinese children and adolescents [47,48]. However, few studies examined the association of attachment insecurity (avoidance and anxiety) with empathy among Chinese adolescents. Additionally, few studies have examined the mediating effect of different aspects of empathy on the association between attachment insecurity and altruistic behavior among Chinese adolescents.

### 1.5. The Present Study

Although previous studies reported that attachment insecurity was related to altruistic behavior, few studies have investigated the differential predictive effects of attachment avoidance and attachment anxiety on altruistic behaviors. Moreover, few studies have examined the mechanisms underlying the association between attachment avoidance as well as attachment anxiety and altruistic behaviors. Furthermore, most studies in this field have been conducted in Western contexts. Therefore, the present study investigated the associations of attachment avoidance and attachment anxiety with altruistic behavior among Chinese adolescents, and examined the mediating effects of different dimensions of empathy (i.e., empathic concern, perspective taking, and personal distress) on the linkage between these two domains. The present study attempted to answer four research questions:

1. Are attachment avoidance and attachment anxiety differentially related to altruistic behavior among Chinese adolescents? Based on the existing Western literature [13,15], we proposed that attachment avoidance would be negatively linked to altruistic behavior (Hypothesis 1a), while attachment anxiety would not be associated with altruistic behavior (Hypothesis 1b).
2. Are there any relationships between attachment insecurity indexed by avoidance and anxiety and different aspects of empathy among Chinese adolescents? Based on the existing Western literature [25,27], we proposed that attachment avoidance would be negatively associated with empathic concern and perspective taking (Hypotheses 2a and 2b), while it would not be associated with personal distress (Hypothesis 2c). Moreover, we proposed that attachment anxiety would be positively associated with personal distress (Hypothesis 2d), while it would not be associated with empathic concern and perspective taking (Hypotheses 2e and 2f).
3. Are different dimensions of empathy associated with altruistic behavior among Chinese adolescents? Based on the existing Western literature [19,30,34], we proposed that empathic concern and perspective taking would be positively related to altruistic behavior (Hypotheses 3a and 3b), while personal distress would be negatively related to altruistic behavior (Hypothesis 3c).
4. Do different components of empathy (empathic concern, personal distress, and perspective taking) have different mediating effects in the relationship between attachment avoidance (and attachment anxiety) and altruistic behavior among Chinese adolescents? Based on the literature reviewed above, we proposed that empathic concern and perspective taking would mediate the relationship between attachment avoidance and altruistic behaviors among Chinese adolescents (Hypotheses 4a and 4b), while personal distress would not mediate this relationship (Hypothesis 4c). Specifically, more attachment avoidance would predict less empathic concern and less perspective taking, which in turn would lead to less altruistic behaviors. Moreover, we proposed that personal distress would mediate the relation between attachment anxiety and altruistic behaviors among Chinese adolescents (Hypothesis 4d), while empathic concern and perspective taking would not mediate this relationship (Hypotheses 4e and 4f). Specifically, we predicted that higher attachment anxiety would predict more personal distress, which would result in a lower level of altruistic behavior.

## 2. Method

### 2.1. Participants and Procedure

Early adolescents were recruited from three junior high schools in urban areas in Chengdu City, Sichuan Province, China, with seven to ten classes in Grade 7 and Grade 8 in every school. All students in the sample classes participated in our study. In total, 1060 students from 25 classes in three schools participated our study. We deleted 55 cases which had missing responses to items on attachment styles, empathy or altruistic behavior. There were no significant differences between the included and excluded participants on the key baseline measures and demographic variables. Finally, there were 1005 students (*M* = 12.86 years, 11–16 years old, *SD* = 0.69; 460 students in 7th grade, 545 students in 8th grade; 479 boys, 513 girls; 80% from 2-parent households; 58.3% students’ parents completed a high school education or above) who were predominantly of Han ethnicity. This study was approved by the Research Ethics Committee at the first author’s university. Informed consent was obtained from all participants and their parents in the study.

### 2.2. Measures

#### 2.2.1. Adult Attachment Styles

We measured attachment styles using the revised scale for adolescents from the short form of the Experiences in Close Relationships Scale [49]. The scale included 12 items measuring adolescents’ levels of attachment anxiety (6 items, e.g., “I worry that close others won’t care about me as much as I care about them”) and attachment avoidance (6 items, e.g., “I try to avoid getting too close to my partner”). Each item was assessed by the students on a 7-point Likert-type scale (1= *disagree strongly* … 7 = *agree strongly*). Higher scores indicate a high level of attachment anxiety or attachment avoidance. The reliability and validity of this scale have been reported in a previous study [49]. In the present study, we deleted three items on attachment avoidance because of their low item–total correlations and standardized factor loadings (less than 0.25 in both cases). As a result, the Cronbach’s alphas for the subscale of the 6-item anxiety and 3-item avoidance were 0.64, and 0.69, respectively.

#### 2.2.2. Empathy

Empathy was assessed using the Interpersonal Reactivity Index [22] including three 7-item subscales on empathic concern (EC), perspective taking (PT), and personal distress (PD). The Empathic Concern Subscale measures the tendency to experience feelings of warmth, compassion, and concern for other people. A typical item from this scale is “When I see someone being taken advantage of, I feel kind of protective toward them”. The Perspective Taking (PT) Subscale measures the tendency to adopt the point of view of other people in everyday life. An example item from the PT subscale is “Before criticizing somebody, I try to imagine how I would feel if I were in their place”. The Personal Distress Subscale assesses one’s own feelings of personal unease and discomfort in reaction to the emotions of others. A PD subscale item is “When I see someone who badly needs help in an emergency, I go to pieces”. Each item was rated by students on a 5-point frequency response scale ranging from 1 (disagree strongly) to 5 (agree strongly). Higher scores indicate greater empathic concern, perspective taking, or personal distress. The reliability and validity of this scale were demonstrated in Chinese participants in a previous study [50,51]. In the present study, we ended up deleting three items on empathic concern (one item) and personal distress (two items) because of their low item–total correlations. As a result, the Cronbach’s alphas for the subscales were 0.69 (EC), 0.72 (PT), and 0.74 (PD), respectively.

#### 2.2.3. Altruistic Behavior

Altruistic behavior was measured using the Altruism Subscale of the Prosocial Behavior Scale developed by Yang et al. [52]. The subscale includes four items (e.g., “I am happy to donate money and materials to the disaster area”. Each item was rated on a 7-point frequency response scale ranging from 1 (disagree strongly) to 7 (agree strongly). Higher scores indicate greater altruistic behaviors. The reliability and validity of this scale were demonstrated in a previous study [52]. In the present study, the Cronbach’s alpha for the subscale was 0.75.

### 2.3. Data Analytic Strategy

First, descriptive statistics and correlation analyses were conducted for the variables of interest for the total sample. Second, we examined the effects of attachment avoidance and attachment anxiety on altruistic behavior. Third, we examined the mediating role of empathic concern, perspective taking, and personal distress in the association of attachment anxiety and avoidance with altruistic behaviors using structural equation modeling (SEM) involving latent variables to minimize measurement errors. When controlling for gender in the models, results were not different from those when gender was not controlled, so gender was not controlled in SEM.

Mplus 7 was used to fit the proposed models to the data [53]. Indirect effects were examined using the bootstrapping method, which allows for hypothesis testing as well as calculation of effect sizes [54]. To evaluate the fit of the models to the data, we examined several standard fit indices, including the comparative fit index (CFI), Tucker–Lewis index (TLI), root mean square error of approximation (RMSEA), and standardized root mean square residual (SRMR). Good model fit was indicated by values greater than 0.90 for the CFI and TLI and smaller than 0.06 and 0.08 for the RMSEA and SRMR, respectively [55,56].

## 3. Results

### 3.1. Descriptive Statistics and Correlation Analyses

Table 1 shows results on descriptive statistics and correlation analyses for all variables. As predicted, we found that attachment avoidance was negatively related with empathic concern, perspective taking, and altruistic behaviors, and positively related with personal distress. On the other hand, attachment anxiety was negatively related to perspective taking and altruistic behaviors, and positively associated with personal distress. Moreover, both empathic concern and perspective taking were positively linked with altruistic behaviors, while personal distress was negatively linked with altruistic behaviors. Consistent with the literature [57], compared to boys, girls reported higher levels of attachment anxiety, and empathy (on all dimensions).

### 3.2. CFA Analyses on the Latent Variables

The latent variables underlying the SEM model were examined via confirmatory factor analysis (CFA). The nine items left in the attachment style questionnaire were the indicators of the latent variables of attachment anxiety (six items) and attachment avoidance (three items). The 18 items retained in the Interpersonal Reactivity Index were used as indicators of the latent variables of empathic concern (six items), perspective taking (seven items), and personal distress (five items). Four items were used as indicators of the latent variables of altruistic behaviors. The model on the latent variables based on confirmatory factor analyses (CFA) showed a good fit to the data, χ^2^ (415) = 1065.91, *p* < 0.001, CFI = 0.909, TLI = 0.900, RMSEA = 0.040 (90% *CI* = 0.037, 0.042), SRMR = 0.051. The factor loadings are presented in Table 2.

### 3.3. Association of Attachment Avoidance and Attachment Anxiety with Altruistic Behavior

The model examined the association of attachment avoidance and attachment anxiety with altruistic behavior and showed a good fit to the data, χ^2^(62) = 206.54, *p* < 0.001, CFI = 0.945, TLI = 0.931, RMSEA = 0.048 (90% *CI* = 0.041, 0.056), SRMR = 0.039. Results indicated that attachment avoidance negatively predicted altruistic behaviors, β = −0.386, *SE* = 0.105, *p* < 0.001, whereas attachment anxiety did not predict altruistic behaviors, β = 0.117, *SE* = 0.107, *p* = 0.270. This result supported Hypothesis 1a and 1b.

### 3.4. Association of Attachment Avoidance and Attachment Anxiety with Altruistic Behaviors: The Mediating Effects of Different Aspects of Empathy

**Figure 1:**
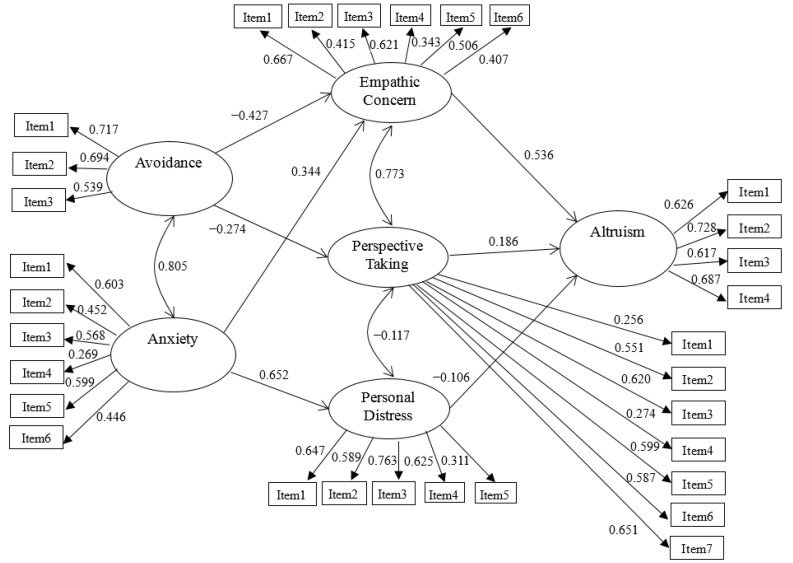
Relations of attachment avoidance and anxiety to altruistic behaviors via empathic concern, perspective taking, and personal distress. *Note*. Only paths with statistical significance (*p* < 0.05) are shown.

The model describing the association of attachment avoidance and attachment anxiety with altruistic behavior via empathic concern, perspective taking, and personal distress (see Figure 1) showed a good fit to the data, χ^2^(416) = 904.94, *p* < 0.001, CFI = 0.915, TLI = 0.905, RMSEA = 0.034 (90% *CI* = 0.031, 0.037), SRMR = 0.052.

Several observations can be highlighted from the findings. First, for the association of attachment avoidance and attachment anxiety with different aspects of empathy, results indicated that attachment avoidance negatively predicted empathic concern and perspective taking, β = −0.427, *SE* = 0.120, *p* < 0.001 and β = −0.274, *SE* = 0.107, *p* = 0.010, respectively, but did not predict personal distress, β = −0.032, *SE* = 0.103, *p* = 0.752, whereas attachment anxiety positively predict empathic concern and personal distress, β = 0.344, *SE* = 0.120, *p* = 0.004 and β = 0.652, *SE* = 0.100, *p* < 0.001, respectively, but did not predict perspective taking, β = 0.005, *SE* = 0.108, *p* = 0.964. This result supported Hypotheses 2a, 2b, 2c, 2d, and 2f, but not Hypothesis 2e.

Second, for the association of different aspects of empathy with altruistic behaviors, results indicated that empathic concern and perspective taking positively predicted altruistic behaviors, β = 0.536, *SE* = 0.086, *p* < 0.001 and β = 0.186, *SE* = 0.087, *p* = 0.033, respectively, whereas personal distress negatively predict altruistic behaviors, β = −0.106, *SE* = 0.054, *p* = 0.048. This result supported Hypotheses 3a, 3b, and 3c.

Third, regarding the test of the indirect effects of empathic concern, perspective taking, and personal distress on the relation between attachment avoidance and altruistic behaviors, results indicated that the mediating effect of empathic concern was significant, β = −0.229, *SE* = 0.074, *p* = 0.002, Bootstrap 95% *CI* = [−0.432, −0.026] (medium effect size) [58]; the mediating effect of perspective taking was marginally significant, β = −0.051, *SE* = 0.030, *p* = 0.091, Bootstrap 95% *CI* = [−0.128, 0.026] (low effect size) [58]; and the mediating effect of personal distress was not significant, β = 0.003, *SE* = 0.011, *p* = 0.761, Bootstrap 95% *CI* = [−0.043, 0.043]. In addition, the direct effect of attachment avoidance on altruistic behaviors was marginally significant, β = −0.162, *SE* = 0.095, *p* = 0.088. This result supported Hypotheses 4a, 4b, and 4c (Table 3).

Finally, regarding the test of the indirect effects of empathic concern, perspective taking, and personal distress on the relationship between attachment anxiety and altruistic behaviors, results indicated that the mediating effect of empathic concern was significant, β = 0.185, *SE* = 0.071, *p* = 0.009, Bootstrap 95% *CI* = [−0.012, 0.381] (low effect size); the mediating effect of personal distress was marginally significant, β = −0.069, *SE* = 0.038, *p* = 0.071, Bootstrap 95% *CI* = [−0.170, 0.032] (low effect size); and the mediating effect of perspective taking was not significant, β = 0.001, *SE* = 0.020, *p* = 0.964, Bootstrap 95% *CI* = [−0.052, 0.054]. In addition, the direct effect of attachment anxiety on altruistic behaviors was not significant, β = 0.065, *SE* = 0.110, *p* = 0.552. This result supported Hypotheses 4d and 4f, but not Hypothesis 4e.

## 4. Discussion

Although previous studies showed that attachment insecurity was related to altruistic behavior, few studies have investigated the differential effects of attachment avoidance and attachment anxiety on altruistic behaviors, particularly in the Chinese context. Moreover, few studies have examined the mediating effects of different dimensions of empathy on the relationship between attachment insecurity and altruistic behavior simultaneously in one study. Furthermore, most studies in this field have been conducted in Western contexts, and had small samples only. Therefore, the present study investigated the relationship between attachment insecurity and altruistic behavior among Chinese adolescents, and examined the mediating effect of different dimensions of empathy. Although we tested many hypotheses in this study, this practice is not uncommon in the scientific literature (e.g., [59,60]).

We found that a high level of attachment avoidance predicted less empathic concern and perspective taking, which in turn predicted less altruistic behaviors. Moreover, we also found a high level of attachment anxiety predicted more empathic concern, which eventually contributed to more altruistic behaviors, while a high level of attachment anxiety predicted more personal distress, which was associated with less altruistic behaviors.

### 4.1. Association of Attachment Avoidance and Attachment Anxiety with Altruistic Behaviors

Results suggested that attachment avoidance could predict altruistic behaviors among Chinese adolescents, whereas attachment anxiety did not predict altruistic behaviors. Our findings are consistent with previous observation that attachment anxiety is an important predictor of anxious reaction to victims, while attachment avoidance is a potentially important predictor of the likelihood of supportive responses to victims [15]. The reason may be that individuals with insecure attachment have negative *internal working models*. In particular, individuals with attachment anxiety have a negative “self-model” with a perception of themselves as unlovable and incompetent, whereas individuals with attachment avoidance have a negative “other-model” with a perception of others as untrustworthy and unreliable, in turn, perceiving others in distress as not deserving of support and help, thereby showing less altruistic behaviors [6,10,11].

### 4.2. Mediating Roles of Empathic Concerns, Perspective Taking, and Personal Distress

Results indicated that a high level of attachment avoidance predicted lower levels of empathic concerns and perspective taking, in turn leading to less altruistic behaviors among Chinese adolescents. This observation supported Hypotheses 4a and 4b and it was consistent with previous studies. Previous studies indicated that attachment avoidance was negatively related to empathic concerns [25], while sympathy (i.e., empathic concerns) tended to be positively related to prosocial behavior [19,30], and sympathy is regarded as the intrinsic motivation of altruistic behaviors [31]. Moreover, according to attachment theory [24], individuals with attachment avoidance may show lower perspective taking behavior which was positively related to prosocial behavior [34]. In summary, attachment avoidance predicts less empathic concern and perspective taking, which in turn predicts less altruistic behaviors. The reason may be that attachment avoidance reflects the degree to which individuals feel uncomfortable with closeness and emotional intimacy in relationships. Those who experience higher attachment avoidance tend to invest less in their relationships and try to remain emotionally independent from their partners. Furthermore, more “avoiding” people tend to rely on “deactivating” attachment strategies, such as suppression of attachment-related thoughts and emotions [8], leading to lower levels of empathic concern and perspective taking.

On the other hand, results indicated that a high level of attachment anxiety predicted more empathic concern, which in turn leads to more altruistic behaviors. This result did not support Hypothesis 4e, and it was contrary to previous findings that attachment anxiety was inversely related to empathic concern [25] or not related to empathy [27]. Moreover, results indicated that a high level of attachment anxiety predicted more personal distress, which in turn predicted less altruistic behaviors. This result supported Hypothesis 4d and was consistent with previous findings that attachment anxiety was positively related to personal distress [25], which in turn was negatively related to prosocial behavior [30]. Taken together, attachment anxiety positively predicted both empathic concern that increased altruistic behaviors and personal distress that reduced altruistic behaviors, which explains why attachment anxiety was not associated with altruistic behaviors. The reason may be that attachment anxiety reflects the degree to which individuals worry and ruminate about being rejected or abandoned by their romantic partners. Those with higher attachment anxiety tend to crave affection from their partners while simultaneously distrusting their partners’ love. Furthermore, more anxious people tend to adopt “hyper-activating” attachment strategies—energetic, insistent attempts to obtain care, support, and love from relationship partners—as a means of regulating distress [8]. As a result, they have more empathic concern and personal distress. Additionally, the positive relationship between attachment anxiety and empathic concern may possibly be a product of collectivistic cultures where people would appear to be “socially desirable” to obtain positive reactions from others—namely, individuals with a higher level of attachment anxiety might show more empathic concern to obtain support from others.

### 4.3. Strengths and Limitations

This study has several strengths. First, this study is the first attempt to explicitly investigate the different effects of attachment avoidance and attachment anxiety on altruistic behaviors among Chinese adolescents. Second, this study suggests that attachment avoidance and attachment anxiety are differentially associated adolescent altruistic behaviors through different paths involving empathic concern, perspective taking, and personal distress. These findings support the attachment theory that individuals with attachment avoidance and attachment anxiety have different internal working models and attachment strategies. Specifically, individuals with attachment anxiety may have a negative “self-model” with a perception of themselves as unlovable and incompetent, and tend to adopt “hyper-activating” attachment strategies as a means of regulating distress, whereas individuals with attachment avoidance have a negative “other-model” with a perception of others as untrustworthy and unreliable, and tend to rely on “deactivating” attachment strategies, such as suppression of attachment-related thoughts and emotions [7,8,10]. Furthermore, these finding also enrich the empathy–altruism connection in the relationship between empathy and altruistic behavior where viewing another’s distress can evoke a mixture of self-focused distress and other-focused empathy [19]. The findings suggest the need to promote parent–child bonding and family functioning to promote positive adolescent developmental outcomes [61,62,63,64].

Despite the above theoretical contributions, there are several limitations of this pioneer study. First, as this study is a cross-sectional study, it is impossible to test the causal relationships because all variables collected in this study are based on a single time point. Second, attachment insecurity was measured using the revised short version of the Experiences in Close Relationships Scale (ECR), which consists of 12 items (6 items for anxiety and 6 for avoidance) [49]. However, three items on avoidance dimension were deleted due to their low item–total correlations and standardized factor loadings. An alternative is that future studies could use the short-revised child version of the ECR (ECR-SRC) [65] to assess adolescents’ attachment insecurity. The Chinese version of ECR-SRC was demonstrated to be valid and reliable in Chinese middle school students [66]. Nevertheless, the present findings showed that the trimmed scales possessed acceptable psychometric properties. Finally, in future studies, to avoid shared method variance and reporter bias, it is necessary to use parent- or peer-reported versions of the scales to measure adolescent behavior.

### 4.4. Implications for Clinical Practice

The present findings have implications for intervention efforts surrounding early adolescents’ attachment insecurity and altruistic behaviors. Specifically, the finding emphasizes the importance of developing programs to improve attachment security via reducing attachment avoidance targeting altruistic behaviors. Attachment quality is a significant concern in China, particularly for parents who are migrant workers (e.g., [67,68]). Theoretically, attachment security (i.e., bonding to parents for adolescents) is a positive youth development (PYD) attribute [69,70]. Empirically, previous studies have suggested that the Project P.A.T.H.S. (Positive Adolescent Training through Holistic Social Programs) could promote positive development in Chinese adolescents [71,72,73,74,75]. Thus, the Project P.A.T.H.S. could be used to promote adolescents’ attachment security, in turn, improving adolescents’ altruistic behaviors.

Moreover, empathic concern and perspective taking could be two important factors linking attachment avoidance to altruistic behaviors, while empathic concern and personal distress could be two important factors linking attachment anxiety to altruistic behaviors. These findings emphasized the importance of developing programs to promote adolescents’ empathic concern and perspective taking, and reduce personal distress targeting altruistic behaviors among adolescents. Likely, empathy is an important characteristic of positive youth development (PYD) [69,70]; the Project P.A.T.H.S. could be applied to promote adolescents’ empathy ability, in turn, improving adolescents’ altruistic behaviors.

Under COVID-19 conditions, how to prevent health behavior (e.g., vaccination) is an important issue to be considered [76]. Besides personal factors such as beliefs and self-efficacy about preventive health behavior, there are views suggesting that empathy plays another role in preventive health behavior (i.e., vaccinate for the sake of protecting others) [77]. In conjunction with the present findings, we may also consider promoting the attachment quality of the public so that they will be more motivated to engage in preventive health behavior [78].

## 5. Conclusions

Overall, our findings suggest that attachment avoidance, not attachment anxiety, could negatively predict adolescents’ altruistic behaviors among Chinese adolescents. Moreover, attachment avoidance predicts less empathic concern and perspective taking, in turn, predicting less altruistic behaviors, while attachment anxiety predicts more empathic concern and personal distress, resulting in more and less altruistic behaviors, respectively.

## Figures and Tables

**Table 1:** Means, standard deviations, and bivariate correlations of attachment avoidance, attachment anxiety, empathic concern, perspective taking, personal distress, and altruistic behaviors.

Variables	*M*	*SD*	1	2	3	4	5	6
1 Attachment avoidance	9.25	4.54	—					
2 Attachment anxiety	19.04	6.82	0.51 ***	—				
3 Empathic concern	22.63	4.21	−0.12 ***	0.02	—			
4 Perspective taking	25.13	4.82	−0.23 ***	−0.19 ***	0.47 ***	—		
5 Personal distress	14.22	4.21	0.37 ***	0.46 ***	0.03	−0.22 ***	—	
6 Altruistic behaviors	22.58	4.42	−0.23 ***	−0.13 ***	0.47 ***	0.46 ***	−0.15 ***	—
7 Gender	*N*_girl_ = 513, 51.7%		0.05	−0.10 **	−0.13 ***	−0.14 ***	−0.18 ***	−0.12 ***

*Note*. Gender was coded with 0 = girl; 1 = boy. ** *p* < 0.01, *** *p* < 0.001. *N* = 1005.

**Table 2:** All variable items and factor loadings.

	Factor Loadings
**Attachment avoidance**	
I want to get close to my partner, but I keep pulling back.	0.718
I am nervous when partners get too close to me.	0.694
I try to avoid getting too close to my partner.	0.538
**Attachment anxiety**	
I worry that romantic partners won’t care about me as much as I care about them.	0.603
My desire to be very close sometimes scares people away.	0.457
I need a lot of reassurance that I am loved by my partner.	0.570
I do not often worry about being abandoned.	0.271
I find that my partner(s) don’t want to get as close as I would like.	0.606
I get frustrated if partners are not available when I need them.	0.441
**Empathic concern**	
I often have tender, concerned feelings for people less fortunate than me.	0.669
Sometimes I don’t feel sorry for other people when they are having problems.	0.411
When I see someone being taken advantage of, I feel kind of protective toward them.	0.618
When I see someone being treated unfairly, I sometimes don’t feel very much pity for them.	0.341
I am often quite touched by things that I see happen.	0.509
I would describe myself as a pretty soft-hearted person.	0.410
**Perspective taking**	
I sometimes find it difficult to see things from the “other guy’s” point of view.	0.255
I try to look at everybody’s side of a disagreement before I make a decision.	0.549
I sometimes try to understand my friends better by imagining how things look from their perspective.	0.619
If I’m sure I’m right about something, I don’t waste much time listening to other people’s arguments.	0.273
I believe that there are two sides to every question and try to look at them both.	0.597
When I’m upset at someone, I usually try to “put myself in his shoes” for a while.	0.586
Before criticizing somebody, I try to imagine how I would feel if I were in their place.	0.650
**Personal distress**	
In emergency situations, I feel apprehensive and ill-at-ease.	0.650
I sometimes feel helpless when I am in the middle of a very emotional situation.	0.587
Being in a tense emotional situation scares me.	0.762
I tend to lose control during emergencies.	0.625
When I see someone who badly needs help in an emergency, I go to pieces.	0.307
**Altruistic behavior**	
I offered to give my seat to someone in need, such as “the old, the sick, and the pregnant”.	0.625
When my classmate fell ill, I sent him to the school doctor’s office.	0.727
I will help my classmates make up lessons or teach them to play basketball.	0.616
I am happy to donate money and materials to the disaster area.	0.686

*Note*. All items and factor loadings are significant (*p* < 0.001). Good model fit was found for all variables on CFA, χ^2^ (*df* = 415) = 1065.91, *p* < 0.001, CFI = 0.909, TLI = 0.900, RMSEA = 0.040, 90% CI = [0.037, 0.042], SRMR = 0.051.

**Table 3:** Standardized indirect effects of attachment avoidance and anxiety on altruistic behaviors via empathic concern, perspective taking, and personal distress.

Indirect Effect	β (Standardized Indirect Effect)	*SE*	*p*	95% *CI* Standardized Indirect Effect
Attachment avoidance and altruistic behaviors				
via Empathic concern	−0.229	0.074	0.002	−0.432, −0.026
via Perspective taking	−0.051	0.030	0.091	−0.128, 0.026
via Personal distress	0.003	0.011	0.761	−0.043, 0.043
Attachment anxiety and altruistic behaviors				
via Empathic concern	0.185	0.071	0.009	−0.012, 0.381
via Perspective taking	0.001	0.020	0.964	−0.052, 0.054
via Personal distress	−0.069	0.038	0.071	−0.170, 0.032

## Data Availability

The datasets generated and/or analyzed during the current study are available from the first author on reasonable request.
